# Mental distress during the COVID-19 pandemic of female students adults without a pre-existing mental health condition

**DOI:** 10.4314/ahs.v21i4.7

**Published:** 2021-12

**Authors:** Marina Vukotic, Zoran Milosevic, Dusko Bjelica, Miodrag Zarubca

**Affiliations:** 1 Faculty for Sport and Physical Education, University of Montenegro; 2 University of Novi Sad, Faculty of Sport and Physical Education, Novi Sad, Serbia; 3 IT center at University of Montenegro

The introduction of socially restrictive measures due to COVID-19 forced students to adapt to new living conditions and take active action. As a consequence of limited movement there was a danger of and increased mental health. It can become worrying if the pandemic of mental health and sedentary lifestyles continues, as well as the further risk of worsening of the resulting condition due to COVID-19^1^. The impact that fear and stress have on every aspect of life while representing a natural reaction to the global health crisis and confronting individuals with the sensitivities of life, both earlier research^2^ and recent findings suggest that infectious disease epidemics and pandemics COVID-19 can be traumatic experiences for some people^3^ and lead to stress and psychological symptoms.^4^ The authors of this study believe that it is of great importance to timely register female students with an increased risk of mental health order to be able to take preventive action. The aim of the this letter is to determine the changes in habits of female students caused by COVID-19 pandemic with the aim of preventing harmful consequences on their mental and psychological symptoms. The sample of respondents were female (n=360; 22.84±5.30 years of age), Students from University of Split, Croatia. The survey question was completed using Google Forms forms, which are posted on the websites of all organizational units of the University of Split in the period from 4 April 2020 to 17 May 2020. The obtained survey results were automatically exported to the Google spreadsheet. Response analysis was evaluated using Office programs such as Excel and SPSS 21 (SPSS Inc., Chicago, IL, USA) in relation to the relevant percentage values of the frequency of responses obtained. When asked when they listen to reports about the increase in the number of people infected with COVID-19, 53.2% of female students feel concerned. When asked if they breathe fast and feel their heart beating, which has not happened to them before, 56.7% of female students answered that this is happening to them, and when asked if they are worried about their health, which could be affected by COVID-19 virus infection 71.5% answered that they were worried about their health. From the above, we can conclude how much the pandemic COVID-19 have a negative impact on the mental health of female students. Therefore, new mental health requirements should undoubtedly be set in many professions, industries. Approaches like this one can be related to all future pandemic conditions, or conditions which can be life-threatening.
